# A deep learning pipeline to simulate fluorodeoxyglucose (FDG) uptake in head and neck cancers using non-contrast CT images without the administration of radioactive tracer

**DOI:** 10.1186/s13244-022-01161-3

**Published:** 2022-03-14

**Authors:** Anirudh Chandrashekar, Ashok Handa, Joel Ward, Vicente Grau, Regent Lee

**Affiliations:** 1 Nuffield Department of Surgical Sciences, John Radcliffe Hospital, University of Oxford, Level 6, Headley Way, Headington, Oxford, OX3 9DU UK; 2 Department of Engineering Science, University of Oxford, Oxford, UK

**Keywords:** Tomography (X-ray computed), Positron emission tomography, Generative adversarial network, Deep learning, Head and neck cancer

## Abstract

**Objectives:**

Positron emission tomography (PET) imaging is a costly tracer-based imaging modality used to visualise abnormal metabolic activity for the management of malignancies. The objective of this study is to demonstrate that non-contrast CTs alone can be used to differentiate regions with different *Fluorodeoxyglucose* (FDG) uptake and simulate PET images to guide clinical management.

**Methods:**

Paired FDG-PET and CT images (*n* = 298 patients) with diagnosed head and neck squamous cell carcinoma (HNSCC) were obtained from The cancer imaging archive. Random forest (RF) classification of CT-derived radiomic features was used to differentiate metabolically active (tumour) and inactive tissues (ex. thyroid tissue). Subsequently, a deep learning generative adversarial network (GAN) was trained for this CT to PET transformation task without tracer injection. The simulated PET images were evaluated for technical accuracy (PERCIST v.1 criteria) and their ability to predict clinical outcome [(1) locoregional recurrence, (2) distant metastasis and (3) patient survival].

**Results:**

From 298 patients, 683 hot spots of elevated FDG uptake (elevated SUV, 6.03 ± 1.71) were identified. RF models of intensity-based CT-derived radiomic features were able to differentiate regions of negligible, low and elevated FDG uptake within and surrounding the tumour. Using the GAN-simulated PET image alone, we were able to predict clinical outcome to the same accuracy as that achieved using FDG-PET images.

**Conclusion:**

This pipeline demonstrates a deep learning methodology to simulate PET images from CT images in HNSCC without the use of radioactive tracer. The same pipeline can be applied to other pathologies that require PET imaging.

**Supplementary Information:**

The online version contains supplementary material available at 10.1186/s13244-022-01161-3.

## Key points

1. CT-derived radiomic features vary significantly in regions of differing metabolic activity.
2. A deep learning generative adversarial network can be used to simulate FDG uptake.
3. Generated PET images from CT were able to accurately predict clinical outcomes.
4. This method allows for the detection of malignant lesions without PET imaging.

## Introduction

Positron emission tomography (PET) is an imaging modality that can be used to visualise abnormal metabolic activity. This is especially important in biological tissues that do not appear pathological based on their morphology [1]. It is a widely adopted clinical tool for the diagnosis, staging and follow-up for a variety of malignancies (pulmonary nodule [2], melanoma [3], head and neck squamous cell carcinoma [4, 5], etc.). It provides clinicians with a semi-quantitative representation of the treatment’s impact and can be used to guide further treatment [1, 6, 7].

The hallmarks of such malignant tissues are rapid proliferation/angiogenesis, increase in size, local invasion, and distant metastasis [8]. At the molecular level, malignant cells have increased glucose utilisation due to an upregulation of enzymatic activity. As a result, injection of a glucose-based radionuclide, Fluorodeoxyglucose (FDG), can be used to identify these abnormal metabolically active tissues. The rate of uptake of FDG into malignant tissues has been shown to be proportional to its metabolic activity [1]. However, unlike glucose, FDG is not fully metabolised and becomes trapped within active cells. This accumulation of FDG is what is observed in a PET image as a ‘hot spot’ and allows for appropriate lesion identification.

Commonly, PET images are obtained alongside a non-contrast computerised tomography (CT) image to enable the localisation of areas of increased metabolic activity with their underlying anatomic structures. Side-by-side comparison or rigid alignment/registration algorithms of independently obtained PET onto CT images were the first methods implemented [7, 9]. However, PET images display few anatomic landmarks that prevent direct correlation with structural images. Furthermore, variability associated with device, protocol and time-point differences in data acquisition (ex. patient repositioning, in-/voluntary movement) limited comparison. The current gold standard involves using hybrid PET/CT units. These units allow for simultaneous acquisition and intrinsic-fusion of PET and CT images with minimal user interaction. Co-registering functional (PET) and anatomic (CT) information has improved specificity/sensitivity of tumour assessment and been shown to improve clinical confidence in decision-making. This advancement has encourage the acceptance and widespread implementation of functional imaging [4, 9, 10].

Although there are numerous advantages for PET/CT imaging, this technique has limitations. Motion artefact between imaging studies may prevent proper co-registration and decreases the clinical value of the obtained images [1, 7, 11, 12]. Additional limitations include (1) increased radiation exposure, and (2) intrinsic patient variability (ex. basal metabolic rate, radionuclide dose, duration between injection and imaging, etc.) [13].

Malignant tissues at the molecular level are significantly different from healthy tissues, in terms of ultrastructure, tissue organisation and metabolic activity [8]. Here, we hypothesise that the raw data acquired from a non-contrast CT contain sufficient information to differentiate regions with different FDG uptake as that can be detected by the PET scan. We further hypothesise that it is feasible to simulate FDG uptake (i.e. a ‘pseudo-PET scan’) from a non-contract CT scan without the need to inject the radioactive tracer, using generative models. We recently reported a deep learning pipeline, using generative models, for a similar medical image transformation task (simulation of contrast-enhanced computerised tomography (CT) without intravenous contrast injection) [14].

## Materials and methods

### Patient population

In this study, we utilised a collection of paired FDG-PET and CT images of 298 patients with diagnosed head and neck squamous cell carcinoma (HNSCC) prospectively recruited from four different institutions in Quebec, Canada. Vallières et al. [5] utilised this clinical cohort to investigate the impact of radiomic methods for the risk assessment of tumour progression. The complete dataset was made publicly available via The Cancer Imaging Archive (TCIA) at http://www.cancerimagingarchive.net. Details of this study, including patient characteristics and clinical outcomes for each of the patients, are elaborated within the Additional file 1: Methods (Additional file 1: Table S1) and are as published [5]. Standard Uptake Value calculation, image alignment/registration (CT to PET) and tumour segmentation methods used in this study are highlighted in the supplement.

### Radiomic feature extraction from tissue segmentations

**Fig. 1:**
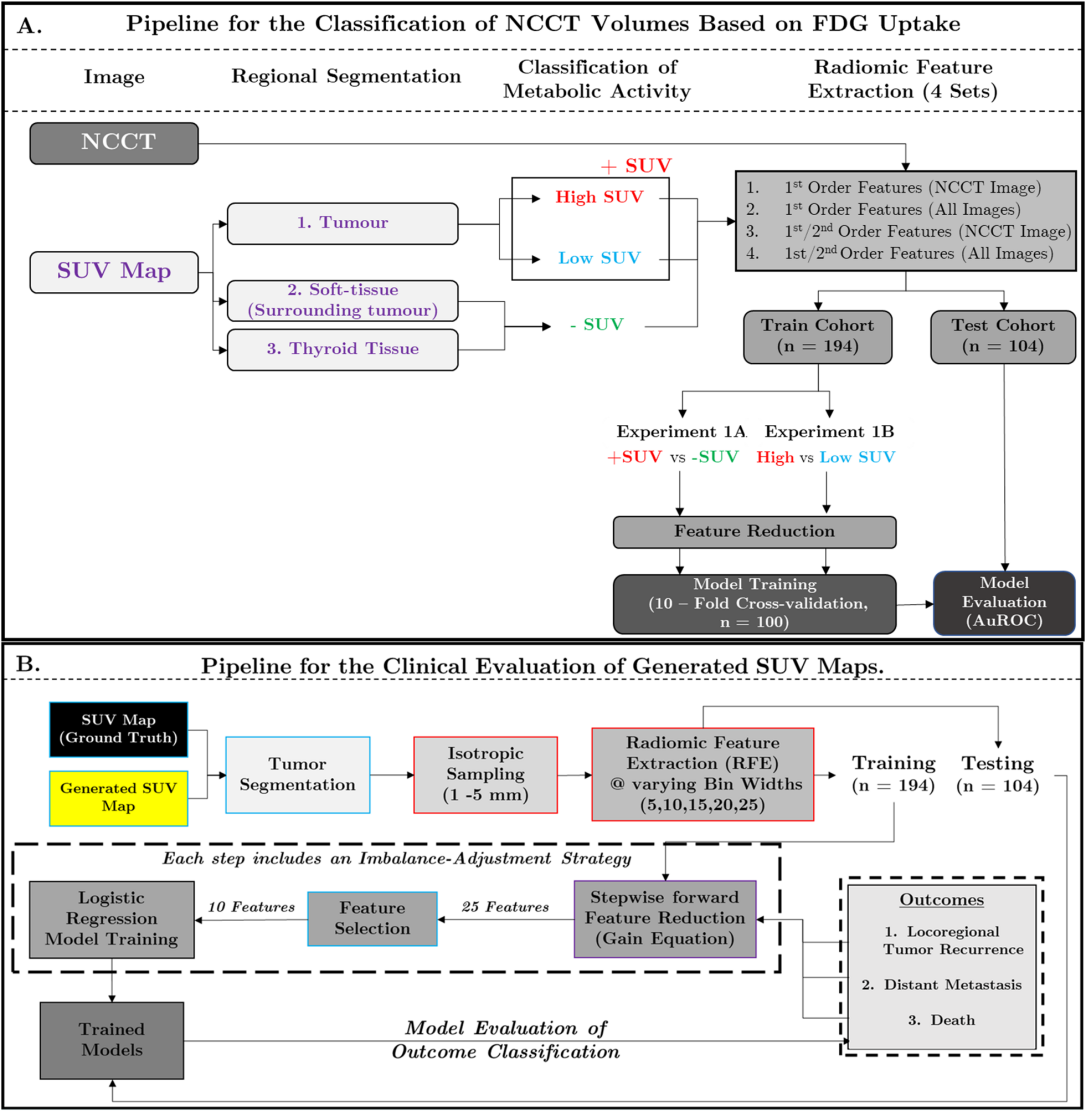
**a** Workflow for the classification of volumes extracted from non-contrast CT images based on FDG uptake using radiomic signature (Experiments 1A and 1B). Tumour and adjacent soft tissue were segmented from the SUV map using threshold-based methods and the thyroid tissue was manually segmented on the paired/registered non-contrast CT image. Volumes were classified based on FDG uptake into three categories (High SUV, Low SUV, and negligible SUV). High/Low SUV were localised within the tumour volume (0.50 × Max SUV). Regions of negligible SUV included the soft tissue surrounding the tumour and thyroid tissue. Four sets of radiomic features were extracted within the segmented volumes from either the CT image or All Images (NCCT + Filtered Images). Filtered images included the CT image with applied Laplacian of Gaussian and Wavelet filters. Full details regarding image filtering can be found within the supplement. Feature reduction was performed in MATLAB using the minimum redundancy, maximum relevance (MRMR) algorithm. Tenfold cross-validation was performed (*n* = 100) and each of the validated models was applied on the testing cohort. **b** Pipeline for the Clinical Evaluation of Simulated SUV Maps. This pipeline is based on the work performed by Vallières et al. which focused on the application of SUV maps for the prediction of three clinical outcomes: (1) Locoregional tumour recurrence, (2) Distant Metastasis and (3) Death. The pipeline consists of radiomic feature extraction from the tumour regions within the SUV Map. Feature reduction, selection and model training were performed on the training cohort using an imbalance-adjustment strategy that was identical to Vallieres et al. Optimised models were evaluated on the testing cohort

Anisotropic image and segmentation masks were resampled into isotropic-sized voxels (Image settings/voxel size: 1 mm, 2 mm, 3 mm, 4 mm and 5 mm) in MATLAB. Parameter settings for radiomic feature extraction included five pre-defined histogram bin widths in Hounsfield Units (5, 10, 15, 20 and 25 HU). All radiomic features were extracted using Pyradiomics, an open-source python package [15]. For each set of image and parameter settings, 18 first-order, 68 s-order and 1118 filter-based features were calculated. This results in a total of 30,100 features for each region-of-interest (ROI) ((86 first-/second-order features + [86 * 13 filtered images]) × 5 Isotropic Settings × 5 Bin-width setting). Detailed information regarding radiomic features extraction can be found within the supplement. The full pipeline for Experiments 1A and 1B is illustrated in Fig. 1a.

In *Experiment 1A*, radiomic features were extracted from tumours with increased FDG uptake and regions with negligible/low FDG uptake [(1) adjacent to tumour and (2) thyroid tissue]. Similarly, in *Experiment 1B*, radiomic features from regions of high and low FDG uptake *within* each metabolically active tumour were extracted. Following feature extraction, patients were divided into training (*n* = 194) and testing (*n* = 104) cohorts. This split was identical to that performed by Vallieres et al. [5]. Given that each patient may have multiple tumour hot spots, train and test cohorts were divided based on patient to prevent data leakage. This was important to prevent different tumour hot spots from a single patient appearing in both the training and testing cohorts. Feature selection, model training and optimisation were performed on the training cohort. The testing cohort was introduced to evaluate model performance.

### Radiomic feature reduction

For *Experiments 1A and 1B*, four different models were trained using a different combination of radiomic features (× Image Settings(5) × Parameter Settings(5)). These models include:

I. First-order features from the CT image
II. First-order features from the CT + filtered CT images
III. First-/second-order features from the CT image
IV. First-/second-order features from the CT + filtered CT images

For each model, features were ranked using the minimum redundancy, maximum relevance (MRMR) algorithm in MATLAB. The top 25 features for each model were selected for model training and optimisation. A detailed explanation regarding radiomics feature extraction for *Experiments 1A* and *1B* is in the supplement.

### Random forest classification of FDG uptake based on radiomic signatures

For each experiment, models I–IV were trained on the training cohort of 194 patients with the appropriate feature set using a tenfold cross-validation approach. Prediction performance was estimated on the testing cohort using receiver operating characteristic (ROC) curves analysis. Area under the ROC (AuROC) was calculated to compare model performance.

### Generative models: non-contrast CT to SUV image transformation

#### Deep learning architecture and model training

A generative adversarial network was used for this non-contrast to SUV image transformation task. These networks are a class of deep learning (DL) architectures whereby two neural networks train simultaneously, with one network focused on data generation (generator) and the other focused on data discrimination (discriminator). These networks compete against each other to better learn the underlying statistical distribution of the training data. Here, we implement a Cycle-GAN, which learns transformations between two distributions without the need for direct pairings between samples. We had previously applied the Cycle-GAN architecture for the simulation of contrast enhancement for CT images [14].

Specifics regarding model architecture and associated training details are described in the Additional file 1: methods*.* A threefold cross-validation paradigm with a training/test data split of 200:98 patients (~ 8400: ~ 3900 2D axial slices) was employed. Model performance during training and validation was evaluated using root-mean-square-error (RMSE) difference between the generated and the ground truth SUV map. This metric is widely used in image transformation tasks as it evaluates the pixel-to-pixel differences between image pairs.

#### Model evaluation: technical assessment of cycle-GAN-generated SUV map accuracy

Tumours within the Cycle-GAN-Generated SUV maps were segmented using the same threshold-based segmentation criterion as used for the ground truth SUV maps. Technical accuracy of the generated SUV maps was assessed by extracting criteria supported by the PET Response Criteria in Solid Tumours (PERCIST, version 1), which is used to monitor tumour progression and response to treatment [16]. For each patient, four clinically important metrics were extracted and compared against that of the ground truth: (1) Minimum SUV(SUV_0_), (2) SUV at the 50th percentile (SUV_50_), (3) Maximum SUV (SUV_Max_) and (4) Tumour burden/volume (in mm^3^). Bland–Altman plot and correlation coefficient analysis were performed for each testing fold to compare the values obtained from the generated SUV map and that from the ground truth SUV map.

#### Model evaluation: clinical outcome prediction using simulated SUV maps

Using the simulated SUV maps, random forest models were constructed to predict three clinical outcomes [(1) locoregional tumour recurrence, (2) distant metastasis, (3) survival]. The primary objective of this experiment was to compare predictive accuracy between the Cycle-GAN-simulated and ground-truth (GT) SUV maps. This analysis mirrors that performed by Vallières et al. [5] and is visualised in Fig. 1b.

For each patient, a total of 2150 radiomic features were extracted. It is important to note that for this experiment, tumour hot spots were grouped by patient and not individually analysed, as was done in Experiments 1A and 1B. Identical training (*n* = 194) and testing splits (*n* = 104) were implemented for model training and evaluation. The process of integrating the radiomic features into a multivariable model was achieved using the logistic regression utilities of the software DREES [5, 17]. Stepwise feature set reduction and selection methods were implemented. Additional information regarding the feature set reduction and selection can be found in the additional files. For each set of features, predictive performance was estimated and the top three parsimonious models were chosen for each outcome. The selected parsimonious models for each feature set [(1) GT-SUV map, and (2) Cycle-GAN-Simulated SUV Map] and each of the three outcomes were directly tested on the pre-defined testing set. Model performances between the generated SUV maps and that of the GT were compared to assess the predictive capacity of the generated images.

## Results

### Patient population and SUV map characteristics

Imaging (PET, CT) data from 298 patients with diagnosed HNSCC were available on TCIA. Standard Uptake Value maps were generated from the provided PET images to standardise measurements between patients. From 298 patients, 683 hot spots of elevated FDG uptake (elevated SUV, 6.03 ± 1.71) were segmented, which represent metabolically active tumours (primary and/or metastatic lymph nodes). These derived segmentations serve as the ground truth for subsequent experiments. Additional information regarding the patient cohort characteristics and SUV map conversion can be found within the supplement and the previously published data documentation [5].

### *Experiment 1A*: radiomic features in CT images can differentiate regions of elevated versus negligible FDG uptake

SUVs were significantly higher within the tumour when compared against non-tumour tissue (6.03 ± 1.7 vs. 3.21 ± 1.00, *p* < 0.001, Additional file 1: Fig. S3a). In the CT images, the average Hounsfield unit (HU) intensity within the tumour was less than that of the adjacent non-tumour tissue, as seen in Additional file 1: Fig. S4b–c (*p* < 0.01). This suggests that there may be a difference, albeit subtle, in the HU distribution between the two regions.

**Fig. 2:**
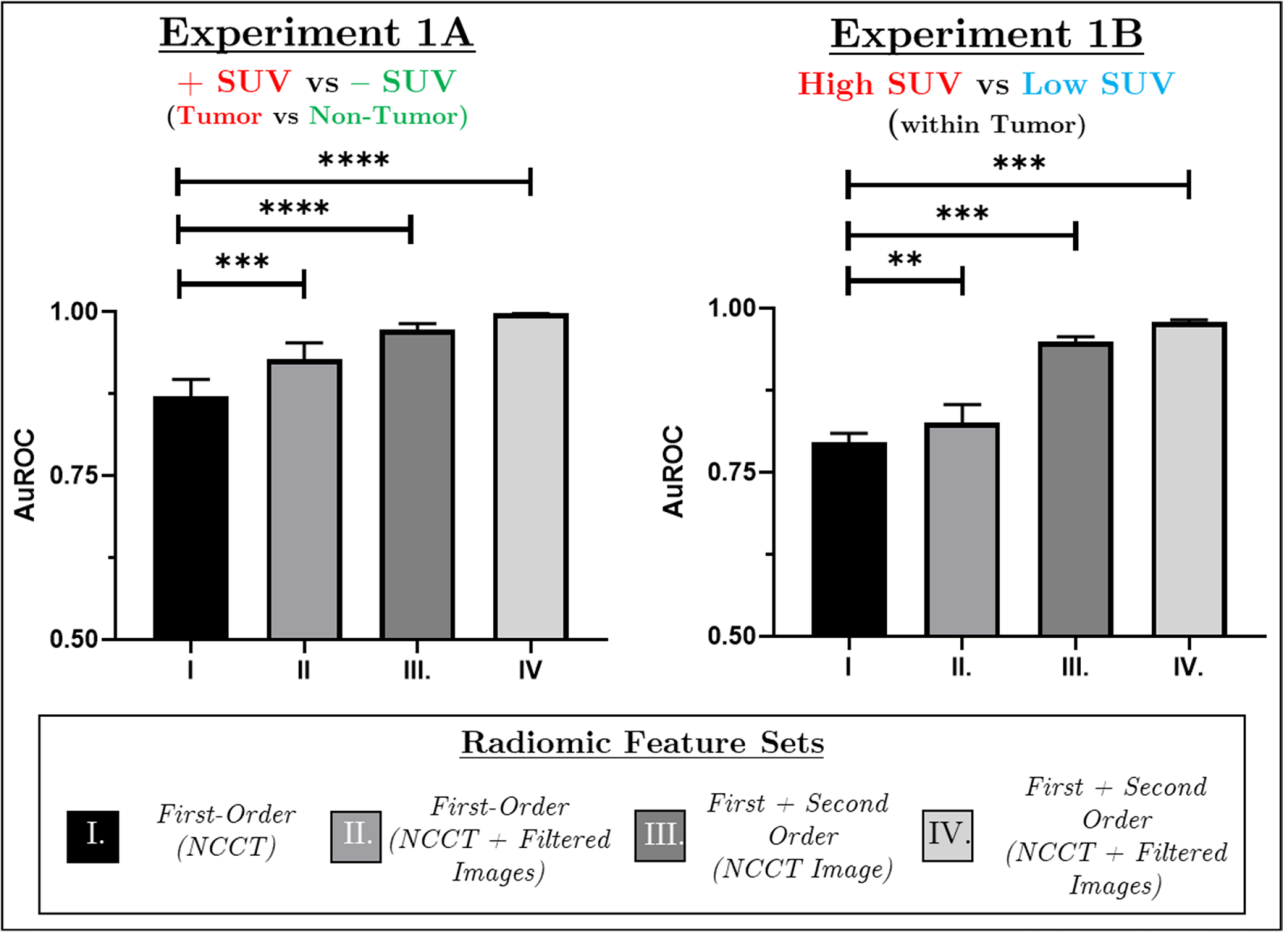
Area under receiver operation curves for four random forest models trained with a combination of radiomic features to classify CT regions based on FDG uptake. Experiment 1A compared regions of elevated versus negligible FDG uptake. Experiment 1B compared tumour regions of High versus Low FDG uptake. Each model was trained using a tenfold cross-validation method for 100 iterations on a selected group of 25 radiomic features. Following training, each of the 100 models is applied to the testing cohort to assess model performance. The statistical differences between each model are assessed using a one-way ANOVA. **p < 0.01; ***p < 0.001; ****p < 0.0001.

Four Random Forest models (*Experiment 1A*: Models I–IV) were trained on a combination of first- and second-order radiomic features extracted from the CT to classify regions with increased or negligible SUV (Fig. 2a). Model I (First Order—CT) had an AuROC of 0.87 ± 0.1 which improved with the introduction of first-order features from filtered images (Model II, First Order—CT + Filter-Based, AuROC—0.93 ± 0.1, *p* < 0.001). The incorporation of matrix-based radiomic features further improved classification performance. Similar differences and model performances were observed when investigating the difference in radiomic signature between tumour and thyroid tissues (Additional file 1: Fig. S5).

### *Experiment 1B*: radiomics features in CT can differentiate high versus low FDG uptake within an individual tumour

A patient-specific SUV_50_ threshold (SUV_50_: 6.62 ± 1.71) was used to further subdivide the tumour into two regions: (1) Regions of high FDG uptake (*n* = 528, SUV: 7.2 ± 2.0) and (2) Regions of low FDG uptake (*n* = 683, SUV: 5.1 ± 1.6, Additional file 1: Fig. S4d). Given that a patient-specific SUV_50_-threshold was used to differentiate regions, tumour hot spots may either have high FDG uptake, low FDG uptake or a combination of the two. Average tumour volume with SUVs above the 50th percentile (8.29 × 10^3^ ± 9.3 × 10^3^ mm^3^) was significantly greater than that below the 50th percentile (6.39 × 10^3^ ± 7.8 × 10^3^ mm^3^, *p* = 0.009). In the CT images, significantly lower HU intensity was observed within the tumour region with higher FDG uptake compared to the tumour regions with lower FDG uptake (*p* < 0.01, Additional file 1: Fig. S4e, f).

Similarly, four random forest models (*Experiment 1B*: Models I–IV) were trained on a combination of first- and second-order radiomic features extracted from the CT to classify regions with high or low FDG uptake within the tumour (Fig. 2b). Model I (First Order—Image-Based) had an AuROC of 0.79 ± 0.13, which improved with the introduction of first-order features from filtered images (Model II, First Order—Image + Filter-Based, AuROC—0.83 ± 0.14, *p* < 0.01). Like that seen for *Experiment 1A*, the incorporation of matrix-based radiomic features (Models III, IV) further improved classification performance.

### Experiment 2: simulation of SUV Map from non-contrast CT

**Fig. 3:**
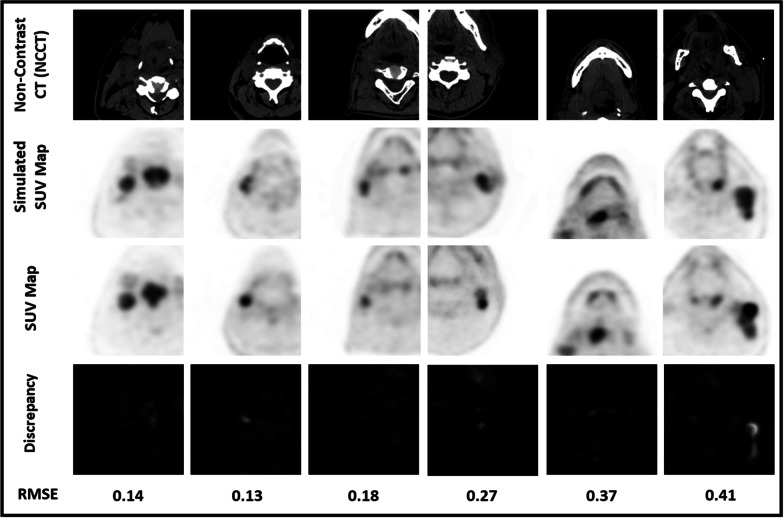
Simulated SUV Map (Output of Cycle-GAN) displayed alongside its ground truth (Real SUV Map) and Non-Contrast CT axial slice for six patients. The error between the SUV maps is visualised and is represented by the RMSE. It is important to note that these SUV maps are inverted as this is the view commonly used by clinicians

A threefold cross-validation platform was implemented for this CT to SUV map image transformation task. During model training, for each fold, the RMSE between the simulated and ground-truth SUV map images for the training and testing cohort decreased to plateau at 0.30 ± 0.12 and 0.40 ± 0.15, respectively (Additional file 1: Table S2). Figure 3 illustrates the generated SUV map alongside their respective gold standards. The visualised error is the difference between the two sets of images and highlights differences in pixel value. The RMSE for each image pair is indicated at the bottom.

#### Technical assessment of simulated SUV map accuracy (PRECIST criteria)

**Fig. 4:**
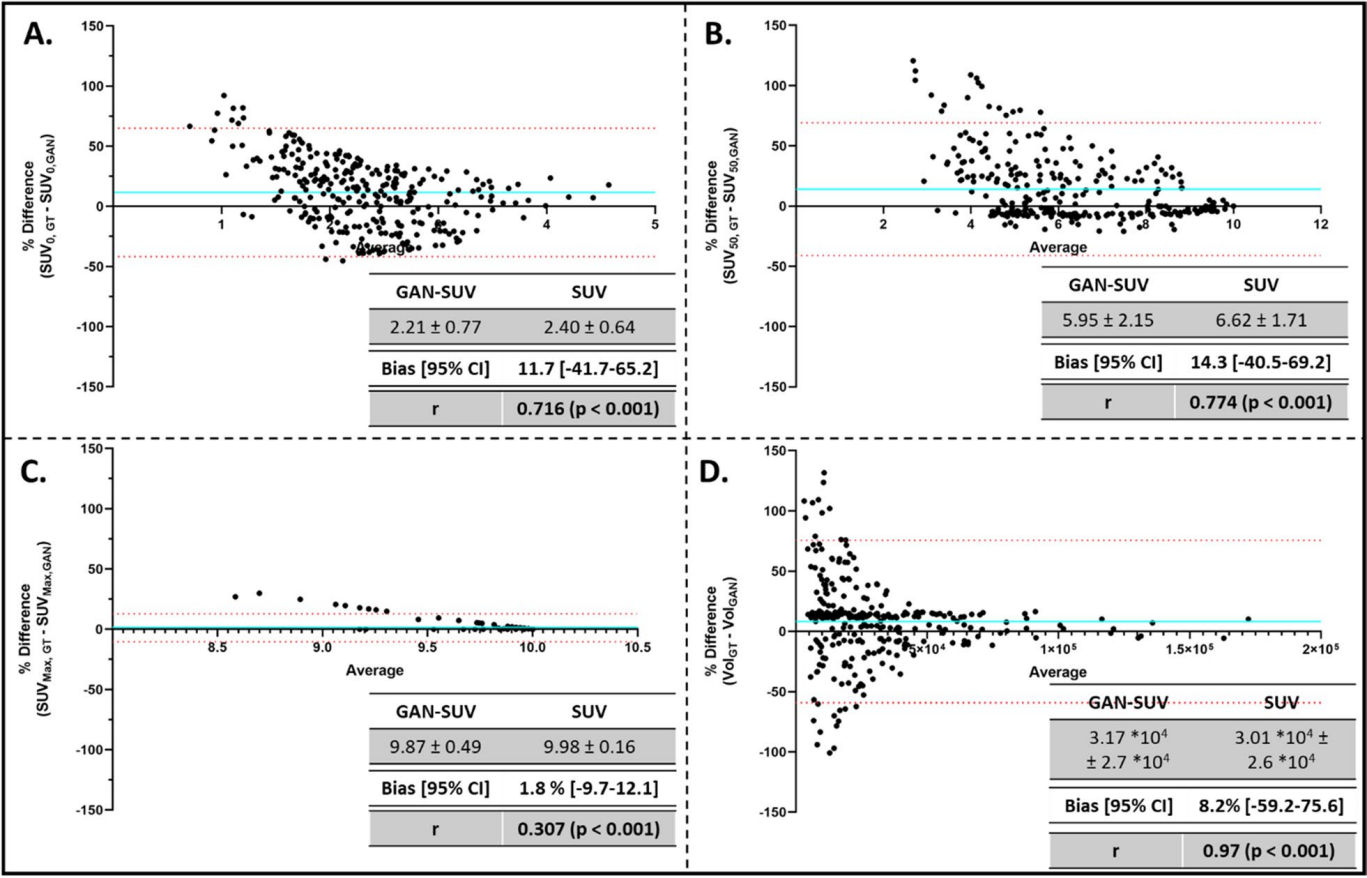
Technical Assessment of Simulated SUV Map Accuracy. Bland–Altman plots for the SUV_0_ (**a**), SUV_50_ (**b**), SUV_Max_ (**c**) and tumour volume (**d**) were constructed to assess the percentage difference between the gold standard and generated SUV maps. The bias along with the 95% confidence intervals is indicated in each plot. These assessment criteria were adapted from the PERCIST v.1 criteria to characterise and monitor tumour progression using PET/SUV images.

Mean SUV_0_ (2.20 ± 0.78), SUV_50_ (5.95 ± 2.15) and SUV_Max_ (9.89 ± 0.38) within the tumour regions of the generated maps were significantly less than that of ground truth (SUV_0_: 2.40 ± 0.64, SUV_50_: 6.62 ± 1.71, SUV_Max_: 9.98 ± 0.15). Subsequently, the bias, as measured by Bland–Altman plot analysis, was 11.7% [95% CI − 41.7 to 65.2%], 14.3% [95% CI − 40.5 to 69.2%] and 1.8% [95% CI − 9.7 to 12.1%], respectively (Fig. 4a–c). These values suggest that the simulated SUV map underestimates FDG uptake within the tumour regions. On the other hand, predicted tumour volume/burden per patient (3.16 × 10^4^ ± 2.73 × 10^4^ mm^3^) was similar to that of the gold standard (3.01 × 10^4^ ± 2.60 × 10^4^ mm^3^, *p* = 0.51). BA plots comparing the percentage differences in tumour burden between the GAN-generated and gold standard SUV MAPs are shown in Fig. 4d.

#### Clinical outcome prediction using simulated SUV maps

Regions of High FDG uptake/SUV were isolated in the Cycle-GAN-simulated SUV (Cycle-GAN-SUV) map using the threshold-based segmentation method. Eighty-six radiomic features (first- + second-order features) were extracted from both the Cycle-GAN-SUV and ground truth-SUV maps (GT-SUV) maps for each combination of image parameters (25). Data were separated into training (*n* = 194) and testing cohorts (*n* = 104) prior to feature reduction and selection for each outcome (Fig. 1b). In all three clinical parameters, there was no difference in the outcome prediction using models generated by GT-SUV maps or Cycle-GAN-SUV maps (Additional file 1: Fig. S6). For the classification of locoregional recurrence, AuROC was 0.60 ± 0.01 (GT-SUV map) and 0.59 ± 0.02 (Cycle-GAN-SUV map) (*p* = 0.35). For classification of distant metastasis, AuROC was 0.82 ± 0.02 (GT-SUV map) and 0.79 ± 0.01 (Cycle-GAN-SUV map) (*p* = 0.20). For the classification of patient death, AuROC was 0.63 ± 0.01 (GT-SUV map) and 0.62 ± 0.02 (Cycle-GAN-SUV map) (*p* = 0.13). A full description of the trained models can be found within the supplement (Additional file 1: Tables S3-S5). The model performances incorporating the GT-SUV maps had similar performance to that observed in the original study by Vallières et al. [5] (AUC, Sensitivity, and Specificity—Additional file 1: Table S6).

## Discussion

We recently demonstrated the feasibility of simulating contrast-enhanced CT images without the injection of IV contrast, using generative DL models. Similar to the workflow described here, we first demonstrated difference in HU intensity and radiomic signature between blood and other soft tissue components [14]. Similarly, abnormal tissues at the molecular level are significantly different from healthy tissues, in terms of ultrastructure, tissue organisation and metabolic activity. These altered characteristics have been shown to be present prior to the alteration in morphological structure at the macro-scale and may reflect changes in the tissue’s attenuation coefficient. We therefore hypothesised that the raw data acquired from a non-contrast CT can be used to identify regions of abnormal metabolic activity.

In general, radiomics employs advanced data characterisation algorithms to extract pixel-based relationships within a pre-defined region-of-interest. In addition to average HU intensity, the differences between these visually in-distinct regions can be captured using a combination of first- and second-order radiomic features. As the first objective, we showed that there are significant radiomic differences between regions of negligible, low, and high FDG activity in the CT image (Experiments 1A-B). These differences support the validity of this image transformation task. The trained DL generative network likely learns this higher-order information during model training.

The second objective was to investigate if a DL generative network could robustly extract the subtle differences between soft tissue components in patients diagnosed with HNSCC and generate a visualisation of FDG uptake. For this task, standard uptake value (SUV) maps were used for the visualisation of FDG uptake. These maps are derived from PET images by standardising for the patient’s weight, radiopharmaceutical dosage (FDG), and the time duration between injection and imaging. These variables account for potential sources of variation within PET imaging.

A threefold cross-validation approach was employed during training/optimisation of the cycle-GAN. There was no data leakage between the training/validation and testing cohorts, ensuring that patients and their respective tumours were either found in the training or testing cohorts. The 2D input data for this DL algorithm were derived from the 3D CT and SUV maps by extracting 2D 144 mm × 144 mm region-of-interests within the larger patient volume. These boundary conditions were defined by the patient contour obtained to evaluate the registration accuracy between the CT and PET images. Additionally, this segmentation was used to remove the underlying table from the patient, especially within the CT image. Given that these scans were obtained from multiple centres, which use PET/CT machines from different manufacturers, the tables that the patients lay on are quite different. Furthermore, as the axial slice moves from the head towards the chest, the 2D axial view of the table significantly changes. Isolating input slices from within the patient volume prevents the generative network from encountering (1) empty slices, (2) slices with a small proportion of the patient and (3) slices with a highly variable table layout. This maximises the information learned by the GAN networks.

We showed that a trained cycle-GAN enables the visualisation of a PET-like output from a routine non-contrast CT without the need to obtain a paired PET image. A subset of the PERCIST criteria was used to evaluate the clinical quality of the generated SUV maps in identifying these tumour hot spots. Volume of the tumour hot spot was similar between the generated and GT-SUV images. This suggests that the generative method is able to differentiate healthy tissues from those with altered FDG uptake. This study shows for the first time the ability to isolate and characterise tumour tissue with altered metabolic activity from the non-contrast CT without the need to inject a radioactive tracer. CT-derived PET visualisation of tumour ‘hot spots’ may serve as a potential inexpensive screening method to select if a patient requires further and more comprehensive PET imaging.

Of note, tumour and non-tumour regions are as defined by the avidity of FDG uptake (SUV) on the PET scan (of the PET-CT). The avidity of FDG uptake (i.e. SUV map on a PET) by a given tissue is proportional to its glucose metabolism. Metabolic activity represents a ‘functional’ status of a tissue and hence the reference as such in this manuscript.

Although the simulated SUV maps tended to underestimate the FDG uptake within the tumour region relative to the GT-SUV maps, they were able to predict clinical outcomes with the same accuracy as the actual PET scan. The methods and analysis for predicting clinical outcome with the GT-SUV maps were adapted from Vallières et al. [5, 17]. Their results identified a combination of radiomic features to predict each clinical scenario. Utilising identical statistical methods for feature reduction and selection, we identify a set of radiomic features that are able to produce similar clinical outcomes within the pre-defined testing cohort. It is important to note that our model performances for locoregional recurrence are slightly higher (AuROC: 0.60) than those presented in Vallières et al. (AuROC: 0.58). The reason for this slight boost in performance falls possibly to the difference in radiomic feature extraction. Our study extracts a total of 2,150 features for each patient (Primary Tumour + Lymph Nodes) − 18 first-order + 68 s-order/texture features for 25 parameter combinations. On the other hand, their study extracted a total of 1615 features from each patient (10 first-order + 5 shape-based + 40 s-order/texture for 40 parameter combinations). Regardless, these results support the ability to use this CT to SUV image transformation method to obtain clinically relevant representations of metabolic activity within patients diagnosed with HNSCCs.

One major limitation of this generative approach that may impact model performance is the variability in the location/pathology of tumours (ex. HPV status) within the head/neck region. This innate pathological variability of the tumours may adversely impact the ability of the generative algorithm in differentiating signal from noise. Other instances of generative networks in medical image transformation tasks are constrained to a more regularly occurring phenotype or pathology [14]. Another limitation is the inherently low resolution of the input non-contrast CT images (CT component of the hybrid PET-CT) as well as differences in imaging protocols between institutions. Improving the accuracy of the generated PET images with regards to its FDG uptake and a 3D implementation is an area of active investigation. That we can derive these results using low resolution CT scans gives further optimism to the full potential of our deep learning approach.

## Conclusion

We present a deep learning pipeline that is able to produce PET-like outputs from non-contrast CT images without the use of radioactive tracer. This generative algorithm captures the quantifiable differences in radiomic signature between regions of different FDG uptake (e.g. Tumours/metastatic lymph nodes vs. thyroid tissue). That such results can be derived using low-resolution CT scans (obtained from historic PET-CT scans) gives further optimism to the full potential of our deep learning approach. The work presented here serves as a primer for further research to reduce/eliminate the need of radioactive tracer injection for cancer imaging.

## Supplementary Information

**Additional file 1:** Supplementary figures and tables.

## Data Availability

Paired FDG-PET and CT images (*n* = 298 patients) with diagnosed head & neck squamous cell carcinoma (HNSCC) were obtained from The Cancer Imaging Archive (https://www.cancerimagingarchive.net/nbia-search/?CollectionCriteria=Head-Neck-PET-CT).

## Abbreviations

AuROC: Area under ROC curve
CT: Non-contrast computed tomography
DL: Deep learning
FDG: Fluorodeoxyglucose
GAN: Generative adversarial network
GT: Ground truth
HNSCC: Head and neck squamous cell carcinoma
HU: Hounsfield unit
PET: Positron emission tomography
RF: Random forest
ROC: Receiver operator characteristic curve
ROI: Region-of-interest
SUV: Standard uptake value
