# Supplementary material for: A deep learning pipeline to simulate fluorodeoxyglucose (FDG) uptake in head and neck cancers using non-contrast CT images without the administration of radioactive tracer
Source: Insights Imaging. 2022 Mar 14;13:45. doi: 10.1186/s13244-022-01161-3 (PMC8921434; doi:10.1186/s13244-022-01161-3)
Supplement: Supplementary file 1 — Additional file 1: Supplementary figures and tables. [file 13244_2022_1161_MOESM1_ESM.docx]

# Additional file 1: Methods

## Patient Population

Retrospective analysis was performed on this imaging/clinical dataset from a total of 298 H&N cancer patients that presented to four different institutions. All included patients received either radiation alone (n = 49, 16%) or chemo-radiation (n = 249, 84%) with curative intent as a part of their routine treatment management. The median follow-up period of all patients was 43 months (range: 6 - 112). Exclusion criteria consisted of patients with 1. Recurrent H&N cancer, 2. Distant metastases at initial presentation, and 3. No observed clinical outcome and a singular follow-up of < 24 months. Patients on palliative care were also excluded. This larger dataset was then divided into training (n = 194) and testing (n = 104) cohorts. This split was identical to that performed in Vallieres et al(*1*). Cohort characteristics are highlighted in **Additional file 1: Table S1**.

## SUV Map Generation from PET Images

As mentioned in the text, Standard uptake value (SUV) is a mathematically derived ratio of tissue radioactivity concentration (A_C_) from the PET image that is standardized to the patient’s body weight (W), the initial radiation dose (D), half-life of radioactive tracer and the time duration between bolus injection and imaging (∆t)(*2*).

| $SUV=\frac{A_{C}}{D* 2^{\left( \frac{-\Delta T}{T_{\frac{1}{2}}} \right)}}*W$ | (Eq. 1) |
| --- | --- |

Although vulnerable to variability (eg.. image noise, low image resolution, region-of-interest-input), this semi-quantitative calculation is a common technique used to standardize PET image comparison between multiple patients and cohorts(*3, 4*). It is important to note that the distribution of pixel values within the 3D images between PET and SUV images are identical apart from the range. Within the SUV image, the range is constrained to between 0 and 10. A sample axial PET and SUV slice are also illustrated, which suggest that this transformation maintains image integrity while shifting the scale.

Additional file 1: Table S1: Patient Characteristics between train/test cohorts.

| **Characteristic** | **Type** | **Train**  **n = 194** | **Test**  **n = 104** |
| --- | --- | --- | --- |
| **Gender** | **Male (%)** | 149 (77) | 80 (77) |
| **Age** | **Mean ± STD** | 63 ± 10 | 63 ± 10 |
| **Tumor Type** | **Oropharynx (%)** | 129 (66) | 77 (74) |
|  | **Hypopharynx (%)** | 5 (3) | 7 (7) |
|  | **Nasopharynx (%)** | 20 (10) | 7 (7) |
|  | **Larynx (%)** | 36 (19) | 9 (9) |
|  | **Unknown (%)** | 4 (2) | 0 (0) |
| **T-Stage** | **T1 (%)** | 29 (15) | 10 (10) |
|  | **T2 (%)** | 65 (34) | 44 (42) |
|  | **T3 (%)** | 66 (34) | 28 (27) |
|  | **T4 (%)** | 30 (15) | 17 (16) |
|  | **Tx (%)** | 4 (2) | 4 (4) |
| **N-Stage** | **N0 (%)** | 51 (26) | 9 (9) |
|  | **N1 (%)** | 29 (15) | 12 (12) |
|  | **N2 (%)** | 108 (56) | 70 (67) |
|  | **N3 (%)** | 6 (3) | 13 (13) |
| **TNM-Stage** | **Stage I (%)** | 4 (2) | 0 (0) |
|  | **Stage II (%)** | 22 (11) | 5 (5) |
|  | **Stage III (%)** | 50 (26) | 13 (13) |
|  | **Stage IV (%)** | 118 (61) | 85 (82) |
| **HPV Status** | **Positive (%)** | 56 (29) | 23 (22) |
|  | **Negative (%)** | 28 (20) | 3 (3) |
|  | **N/A (%)** | 100 (51) | 78 (75) |
| **Treatment** | **Radiation Only (%)** | 38 (20) | 11 (10) |
|  | **Chemo-radiation (%)** | 156 (80) | 93 (90) |
| **Outcome** | **Locoregional recurrence (%)** | 29 (15) | 16 (15) |
|  | **Distant Metastases (%)** | 26 (13) | 14 (13) |
|  | **Death (%)** | 32 (17) | 24 (23) |

## Patient Contour Segmentation from Non-Contrast CT and SUV images and Image Registration

Direct comparison of CT with the PET/SUV images require the two images to be registered and display considerable overlap. This allows for sufficient localization of the PET/SUV map to the anatomical framework provided by the CT image. The clinical value of the subsequent experiments relies on the quality of registration between images. Registration accuracy was assessed by comparing the patient contours obtained from the from the CT and PET/SUV images.

Binary segmentation of the SUV map was generated in MATLAB using threshold-based methods (SUV ≥ 0.1, 1 [patient]; SUV < 0.1, 0 [background]). The SUV threshold of 0.1 was defined as the smallest positive SUV within the map. Subsequent morphological dilatation and erosion operations using a spherical structuring element within the interior of the patient was used to connect components. 3D-gaussian filtering on the segmented images (sigma, 2) was used to smooth all SUV-map derived patient segmentations (**Additional file 1: Fig. S1a**). Similar threshold-based and dilation/erosion methods were used to generate the segmentation from the Non-Contrast CT (Hounsfield Unit, HU ≥ -500, 1 [patient]; HU < -500, 0 [background], **Additional file 1:** **Fig. S1b**). The CT threshold of -500 was defined empirically as it is able to capture all soft-tissue regions within the anatomical CT region. Registration accuracy was assessed using the Sorensen-Dice (DICE) coefficient, which is a ratio comparing the similarity between segmentations (2 * elements common to both images/ the total number of elements, **Additional file 1:** **Fig. S1c**). If the DICE score was less than 90%, a non-rigid b-spline registration algorithm was implemented on the segmentations to ensure registration accuracy. This registration method was used in our previous work(*5*). Finally, this method was used to isolate the patient from the underlying table in the CT (yellow arrows, **Additional file 1: Fig. S1d**).


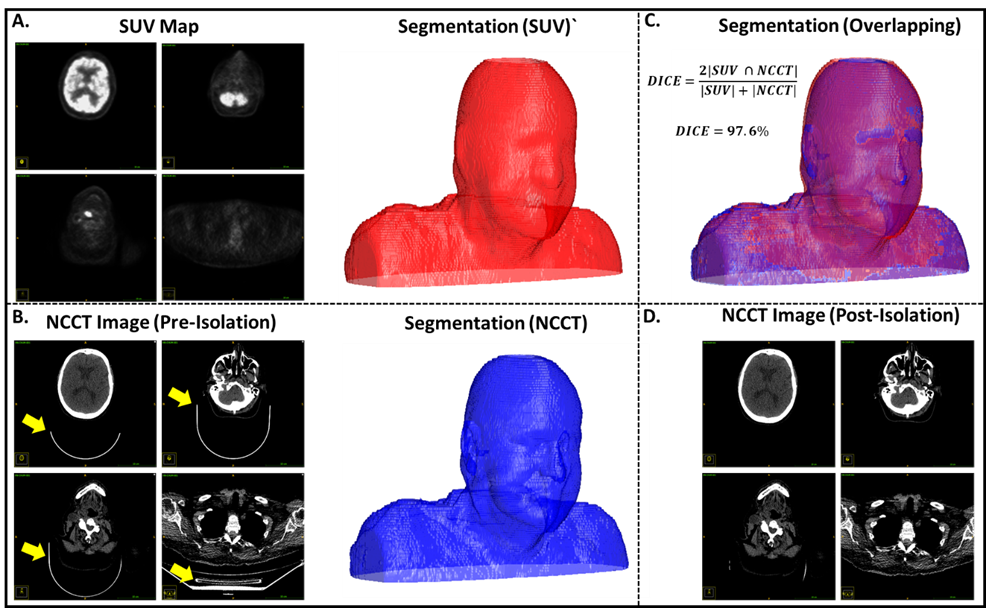


**Additional file 1: Fig. S1: Patient Surface Contour Segmentation from Non Contrast CT and SUV Images and Image Registration.**

**A-B.** The surface contour of each patient’s head was segmented from both the NCCT and SUV using threshold-based methods followed by morphological dilation/erosion using a spherical structuring element in MATLAB. Generated segmentations were smoothed using a gaussian filter (sigma, 2). **C.** Registration accuracy was assessed using DICE score overlap. **D.** Additionally, in the NCCT image, the generated segmentation was used to remove the table (yellow arrow). The post-isolated NCCT and SUV images are input for subsequent investigations.

## Tumour Segmentation of the SUV Map for Radiomic Analysis

The primary focus of this experiment was to characterise and compare the radiomic signatures of highly metabolic tumours with elevated FDG uptake (increased Standard Uptake Value, SUV) against regions of low/negligible FDG uptake. CT and SUV map registration was ensured using b-spline non-rigid registration methods. Tumour segmentation was performed using the PET-derived SUV map. The following assumptions were made: 1. the SUV map and CT image are registered and display good alignment, 2. In the head and neck region, the brain is highly metabolically active, has consistently high FDG uptake and is the largest connected component in the SUV map. Tissue containing tumour are highly metabolic and display increased FDG uptake. A patient-specific threshold (0.35 x Maximum SUV) was empirically defined based on its consistency at differentiating metabolically-active tissue (with elevated SUV, ex tumour) from tissues with negligible activity. This SUV threshold was selected as a conservative approach to isolate metabolically-active regions as it greater than the general SUV threshold suggestive of malignancy (SUV of 2.5)(*6*). A convolution filter with a kernel size of 3 was used to smooth the initial segmentation output (**Additional file 1: Fig. S2a**). Subsequently, connected component analysis was used to separate the brain from other regions of elevated FDG uptake (**Additional file 1: Fig. S2b**). All generated segmentations were assessed for manual overlap with the SUV Map and minor adjustments were made.

***Experiment 1A*** investigated the radiomic differences *between* regions with elevated FDG uptake (+SUV, ie tumour) and regions of low/negligible FDG uptake (-SUV, ie non-tumor). Thyroid tissue was manually segmented and used as a biological comparison. We reasoned that thyroid tissue has inherent metabolic activity and has resemblance to lymph nodes in terms of macroscopic structure.

***Experiment 1B*** characterised the radiomic differences *within* different regions of each of the metabolically-active tumours.

### Segmentation of tumour and non-tumour tissues (Experiment 1A)

*Experiment 1A* aims to investigate the radiomic differences between regions with elevated FDG uptake (+SUV, ie tumour) and regions of low/negligible FDG uptake (-SUV, ie non-tumor). Regions of negligible FDG uptake include tissues immediately adjacent to the segmented tumour region and thyroid tissue. Thyroid tissue was selected as the negative control as it is biological tissue that is similar to non-tumorous lymph nodes, with regards to tissue ultrastructure and in appearance on an CT image. To sample adjacent regions to the tumour, the tumour surface was dilated by a factor of 2. The centroid of the expanded segmentation mask was matched to that of the original segmentation. This resulted in two concentric segmentations with equal volumes (**Additional file 1: Fig. S3a**). Thyroid segmentation was performed by a trained clinician directly on the CT image using an open-source segmentation software, ITK-Snap.

### Regional tumour segmentation based on FDG Uptake (Experiment 1B)

*Experiment 1B* aims to characterise the radiomic differences within different regions of a metabolically-active tumour. Tumours were divided into two sub-regions based on the FDG uptake avidity: 1. High FDG uptake (≥ 50^th^ percentile of SUVs [SUV_50_] within the tumour), and 2. Low FDG uptake (< SUV_50_). The constrained sub-region represents an area of higher FDG uptake within the tumour volume (**Additional file 1: Fig. S3b**). The SUV_50_ was specific to each patient and allowed for the differentiation of FDG uptake within the tumour.


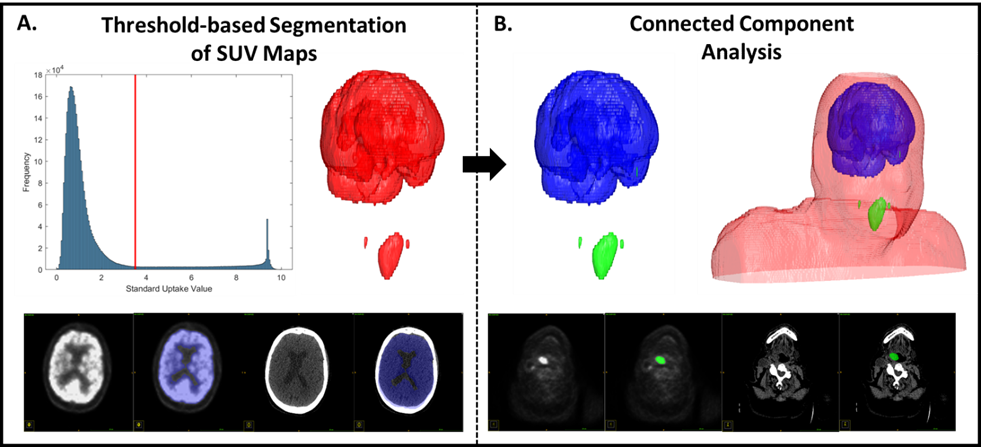


**Additional file 1: Fig. S2: SUV-Map-based Tumour Segmentation for Radiomic Analysis.**

**A.** Threshold Based Segmentation of SUV maps was performed by using an empirically-defined threshold. Segmentations were smoothed using a convolutional filter (kernel size, 3). **B.** Connected component analysis was performed to isolate the brain from other hotspots, which are characteristic of the tumour (primary and/or lymph nodes). The extracted segmentations are visualized along with their respective NCCT image.


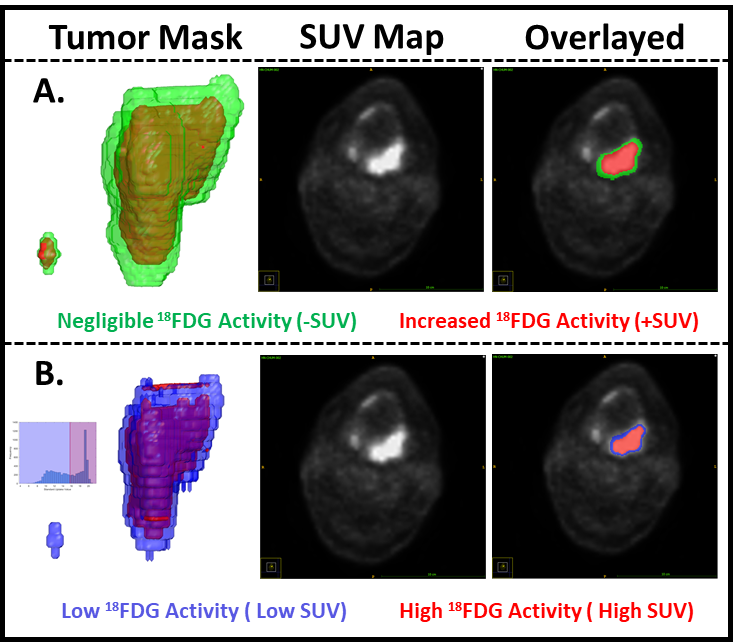


**Additional file 1: Fig. S3: Regions of different FDG uptake avidity as defined by the PET SUV map.**

**A.** For Experiment 1A, the tumour contour (red) was dilated by a factor of 2 at its centroid. The two concentric regions represent areas of elevated FDG uptake (red) and negligible FDG uptake (green), respectively. **B.** For Experiment 1B, the tumour was divided into two sub-regions based on the FDG uptake avidity, using 50^th^ Percentile of SUV (SUV_50_) as the threshold.

### Radiomic Feature Extraction

All radiomic features were extracted using Pyradiomics, an open-source python package(*7*). For each set of image/segmentation and parameter setting, 18 first-order, 68 second-order and 1118 filter-based features were calculated.

1. ***First-Order features:*** These features consist of image-based statistics (ex. minimum, mean, median, maximum, kurtosis, etc), which describe the distribution of voxel intensities within the image region defined by the segmentation mask. These features are not influenced by the shape or volume of the defined mask.
2. ***Second-order features***: These matrix-based features extract the radiomic “texture” within a defined region of interest shape/volume. These features include those derived from 1. Gray-level Co-occurrence (GLCM), 2. Gray-Level Size Zone (GLSZM), 3. Gray Level Run Length (GLRLM), 4. Neighbouring Gray Tone Difference (NGTDM) and 5. Gray level Dependence (GLDM) matrices.
3. **Filter-based features:** These features represent 1^st^ and 2^nd^-order features calculated on filtered images. The two filtering methods applied to the images includes 1. Laplacian of gaussian (LOG) and 2. Wavelet filtering. The former is an edge enhancement filter that seeks to emphasize area of gray level change, where a predefined sigma indicates the degree of coarseness in the filtered image. Here, a high sigma favours coarse textures (grey level variations over a large scale), whereas a low sigma favours finer textures (grey level variations over a smaller scale). In this study, 5 levels of sigma were used (σ = 1,2,3,4,5) with 86 1^st^/2^nd^ order features extracted for each sigma (425 - 3^rd^ order LOG features). Similarly, wavelet filtering produces 8 decompositions for each input image; these decompositions involve applying either a high (H) and/or low (L) pass filter in 3-dimensions (HHH, HHL, HLH, HLL, LLL, LLH, LHL, LHH). For each decomposition, 86 1^st^ and 2^nd^ order features are extracted (680 – 3^rd^ Order Wavelet features). Full documentation for each of the extracted features can be found along with the pyradiomics source code(*7*).

In Experiments 1A-B, first (n = 18), second-order (n = 68) and filter-based (n = 1118) radiomic features were extracted using all the possible combinations (25) of the following parameters:

1. Isotropic voxel size (5): Sizes of 1mm, 2mm, 3mm, 4mm and 5mm
2. Histogram Bin Widths (5): Widths of 5, 10, 15, 20 and 25

For each region of interest, a total of 30,100 features were extracted ((86 1^st^/2^nd^ order features + [86 * 13 filtered images]) x 5 Isotropic Settings x 5 Bin-width settings). When investigating the clinical relevance of the simulated SUV map, only first and second-order features were extracted for each parameter combination (2,150 features per patient, 86 features/combination * 25 combinations). This was done to mimic radiomic features extracted by Vallières et al(*1*).

## Feature Reduction and Selection

Prior to classification of FDG activity in Experiments 1A/1B, feature reduction was performed using the minimum redundancy, maximum relevance (MRMR) algorithm in MATLAB. The top 25 features (from the 30,125 radiomic features per region) for each model were selected for model training and optimization. This algorithm was selected for feature selection as it tends to efficiently parse through a large array of features to select a subset with a high correlation to an output class and a low correlation between the features(*8, 9*). By scaling down the feature vector, it prevents overfitting and maximizes model interpretability, which is extremely valuable for clinical problems.

With regards to clinical outcome prediction, feature set reduction was performed on each initial feature set (2,150 features x 194 patients) in a stepwise forward feature selection method utilizing the Gain equation to obtain a subset of 25 features. These selected features maximized both predictive power (via Spearman’s rank correlation) and non-redundancy (via the maximal information coefficient). Subsequently, feature selection was performed using a stepwise forward feature selection scheme to maximize the 0.632+ bootstrap AUC (100 samples). This resulted in a combination of features (1-10 features) that optimized model performance. For each of the 10 combinations, predictive performance was estimated and the top 3 parsimonious models were chosen for each outcome. Additional detail regarding the feature reduction/selection steps can be found within the Additional file 1 of Vallières et al(*1*).

## Generative Model Training (Non-Contrast-CT to SUV Transformation)

### CycleGAN Architecture

The generator and discriminator components in the Cycle-GAN model architecture were explicitly defined as least-squares GAN and a 70 x 70 pixel PatchGAN, respectively. The former incorporates an additional least-squares loss function for the discriminator, which in turn, improves the training of the generative model. On the other hand, the discriminator goes through the image pairs, in 70 x 70 patches, and is trained to classify whether the image under question is “real” or “fake”. A similar model architecture was used in our prior work for another image medical image transformation task(*5*).

### GAN model training

The Cycle-GAN models were trained with a learning rate of 2.0 * 10^-5^ for 200 epochs on overlapping 144 x 144 images located around the patient segmentation, which was derived to assess registration accuracy between the NCCT and SUV images. Four networks (2 generators + 2 discriminators) were trained simultaneously and various loss functions were evaluated at each iteration to document model training. In addition to the loss metrics inherent to the networks, an identity mapping and a cycle consistency loss functions were included to ensure appropriate style transfer and regularization of the generator to allow for image translation, respectively. Model weights were saved every 10 epochs and intermediate model predictions were simulated from the NCCT images within the training cohort. The simulated predictions were independently evaluated against the ground truth SUV images to assess model training.

Three Cycle-GAN networks were trained on a different set of 200 patients and tested on the remaining 98 patients. Within each training cohort, 50 of the 200 patients were used to internally validate during model training/optimization. The optimized model was then evaluated against the testing cohort. It is important to note that the training/test data split for this experiment are different than previously used (*Experiments 1A/1B*). This data split and training paradigm was used to maximize both the training and testing datasets, allowing all CT/SUV images to be used for training and testing. During assessment, overlapping 144x144 images throughout the patient volume were transformed and a weighted average of the output slices was used to compile the simulated 3D SUV map.

# Additioal file 1: Results

## Patient Population and SUV Map Characteristics

Imaging (PET, CT) data from 298 patients with diagnosed HNSCC were available on TCIA. The patients underwent routine treatment management (radiation – 48, 16%; chemo-radiation – 252, 84%). Imaging was obtained within a median of 18 days (range 6 – 66) prior to the start of treatment(*1*).

At the time of imaging, the median patient weight was 75 Kg (range – 43 -142 Kg) and the median dosage of FDG-PET injected was 1.65 x 10^8^ Bq (range – 3.81 x 10^8^ – 31.82 x 10^8^). Additionally, the median duration between injection and scan time (∆t) was 1.80 x 10^4^ s (range – 1.04 x 10^4^ – 3.01 x 10^4^). Each patient-specific combination of weight, dose, and ∆t, along with the half-life of FDG (6588 s^-1^) was used to calculate the SUV map. Average SUV within the calculated images was 0.19 ± 0.06. SUV maps were derived from the provided PET images to standardize measurements between patients. From 298 patients, 683 hot spots of elevated FDG uptake (elevated SUV, 6.03 ± 1.71) were segmented, which are characteristic of metabolically active tumours (primary and/or metastatic lymph nodes). These derived segmentations would serve as the ground truth for subsequent experiments.

The median follow up time after treatment was 43 months (range – 6 -112 months). Of the 298 patients, 45 patients developed locoregional recurrence, 40 patients developed distant metastasis and 56 patients died. Additional information regarding the patient cohort characteristics can be found within the previously published data documentation(*1*).

## CT/SUV Registration and Tumour Segmentation

The accuracy of registration between the CT and SUV images was 95.1 ± 1.9%. This was assessed by the DICE score between the surface contour of each patient derived from the CT and PET images (**Additional file 1: Fig S1c**).

## Experiment 1A/1B: Radiomic features in CT images can differentiate regions of differing FDG uptake


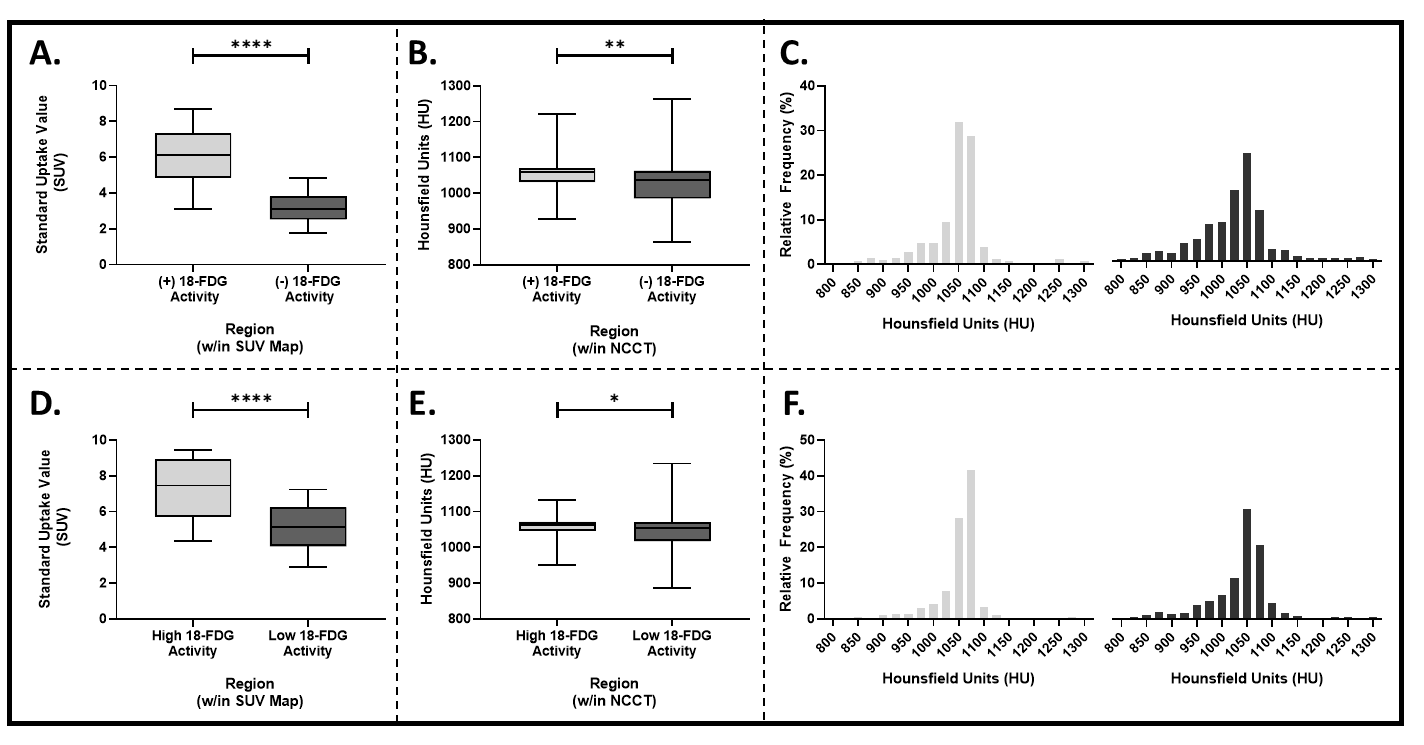


**Additional file 1: Fig. S4: Differentiating regions of FDG uptake within a NCCT image.**

Regions of elevated or negligible FDG uptake (Experiment 1A) had statistically different mean SUVs (A, positive control) and mean Hounsfield Unit intensities (B, p< 0.01). Similarly, segmentations encompassing regions of high and low uptake within the tumor region (Experiment 1B) had statistically different mean SUVs (D, positive control) and mean Hounsfield Unity Intensities (E, p<0.05). This slight difference between regions can be appreciated in the adjacent histograms (C, F).


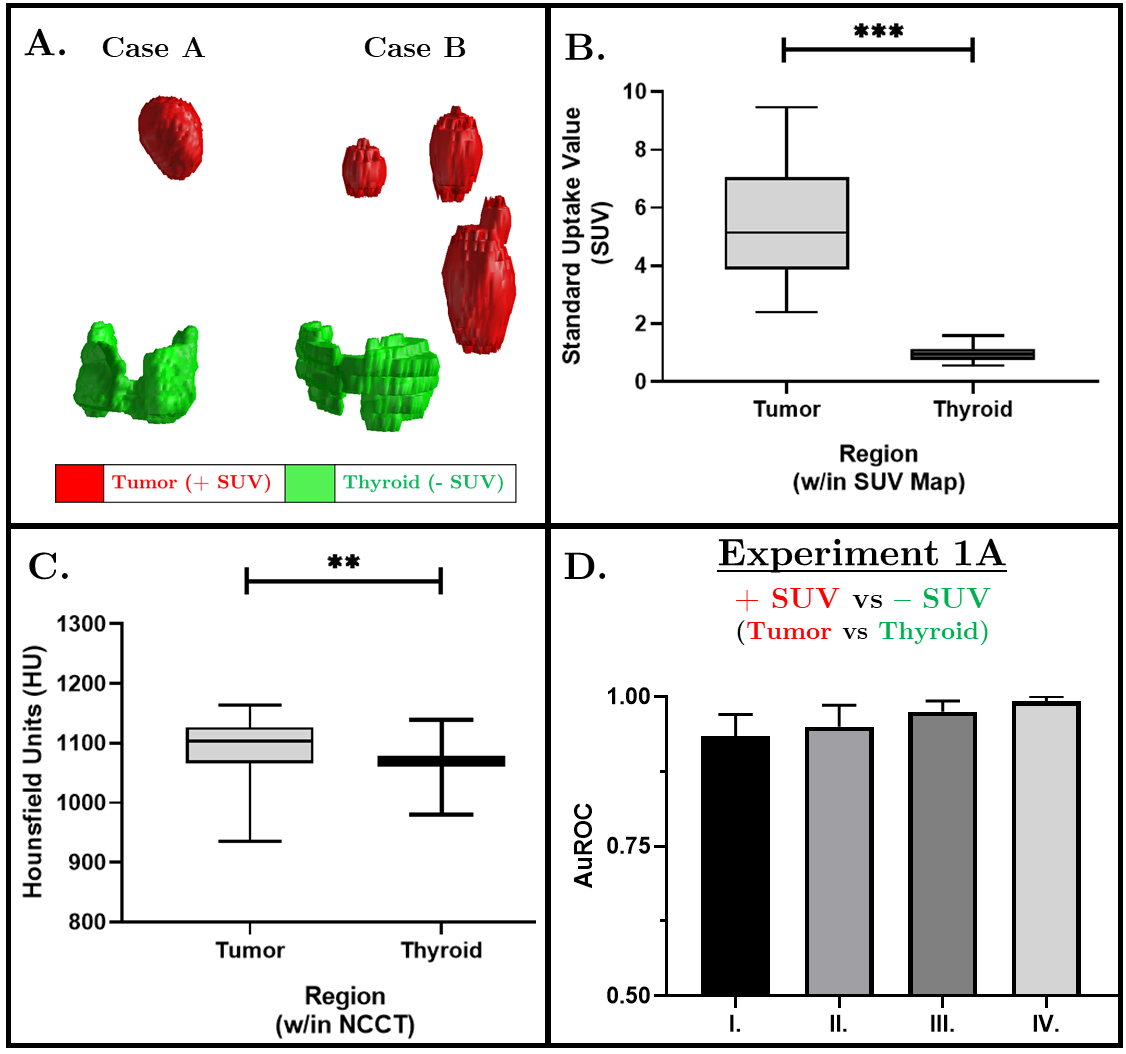


**Additional file 1: Fig. S5: Differentiate tissues (Thyroid vs. Tumour) with different FDG Uptake within a Non-Contrast CT Image. A.** Visualisation of the segmented thyroid (green) and tumour (red) regions from two patients within the dataset. **B-C.** Standard Uptake values (PET/SUV map) and Hounsfield Units (CT) within the tumour region were significantly greater within tumours than that within the thyroid tissue. **D.** Random forest models were trained (10-fold cross-validation, n = 100) using four radiomic feature sets (Models I- IV) to classify the radiomic signature of the region-of-interest based on FDG Uptake. The features sets utilized were I. 1^st^ Order – CT image, II. 1^st^ Order – CT + Filtered Images, III. 1^st^/2^nd^ Order – CT Image and IV. 1^st^/2^nd^ Order – CT + Filtered Images. Identical model frameworks were used for Experiments 1A (Tumour vs non-tumour tissue) and 1B (High vs Low SUV within the Tumour). Area under the Curve for Model I (1^st^ Order radiomic features from CT) was 0.94 ± 0.11. Although model performance increases with feature complexity, the difference in AuROC was not statistically significant.

## Experiment 2: Generation of SUV Maps from CT

**Additional file 1: Table S2:** RMSE difference between GT- and GAN- SUV Maps within the testing cohorts.

| **Test Cohort  in each Fold** | **Patients** | **Tumour**  **hotpots** | **2D Slices** | **RMSE** |
| --- | --- | --- | --- | --- |
| 1 | 100 | 253 | 3,931 | 0.40 ± 0.14 |
| 2 | 100 | 227 | 4,126 | 0.39 ± 0.16 |
| 3 | 98 | 202 | 4,244 | 0.42 ± 0.15 |
| **Total** | **298** | **682** | **12,301** | **0.40 ± 0.15** |

### Outcome prediction using Simulated SUV maps

**
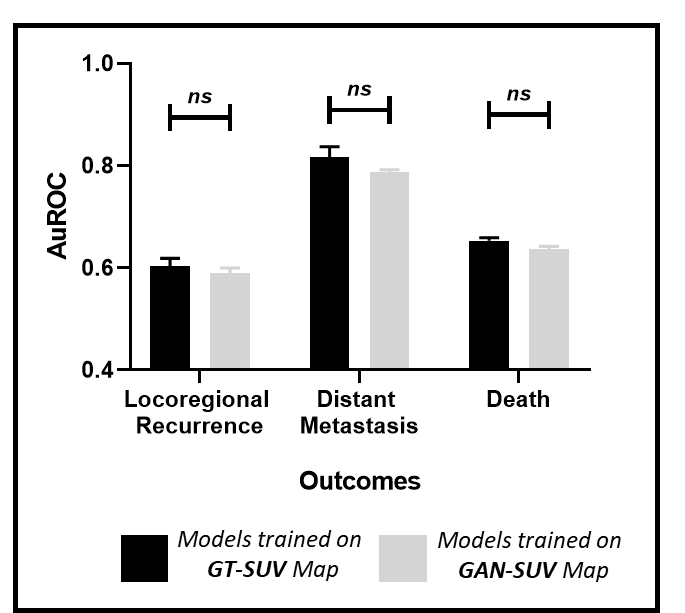
**

**Additional file 1: Fig. S6: Area under ROC curve for logistic regression models trained to predict clinical outcomes**

(1. locoregional tumor recurrence, 2. distant metastasis, and 3. death). Models were trained using selected radiomic features from either the GT- or the CycleGAN- SUV map and evaluated on a fixed testing cohort. The 3 best performing models for each outcome were selected and evaluated. The performance between GT and GAN-SUV maps were analyzed for statistical significance for each outcome investigated. Methods for the GT-SUV map analysis were adapted from from Vallières et al (2017).

This section provides the complete description (specific radiomic features with extraction parameters) of the best radiomic models trained, from both the GT and simulated SUV maps, for each outcome (locoregional recurrence –**Additional file 1: Table S3**, distant metastasis – **Additional file 1: Table S4**, and death – **Additional file 1: Table S5**). Significance of the variable in each of the trained logistic regression models (constructed/optimized from the training dataset - n = 194) was assessed for variable significance using the Wald’s test using the DREES software (MATLAB). **Additional file 1: Table S6** indicates the performance (AUC, sensitivity, specificity and accuracy) of the best logistic regression models for each of the outcomes on the testing dataset (n = 104). Regression models for the simulated and ground truth SUV maps are similar in performance.

**Additional file 1: Table S3:** **Locoregional Tumour Recurrence (all Variables, p < 0.05)**

| Fx | Gold-Standard SUV Map | p | Simulated SUV Map | p |
| --- | --- | --- | --- | --- |
| 1 | ***GLRLM_ShortRunEmphasis:***  Scale = 1mm , binWidth = 25 | ***0.04*** | ***GLCM_Imc1:***  Scale = 5mm , binWidth = 25 | ***0.02*** |
| 2 | ***GLSZM_SmallAreaEmphasis:***  Scale = 2 mm, binWidth = 15 | ***0.008*** | ***GLSZM_ZonePercentage:***  Scale = 1 mm, binWidth = 20 | ***0.005*** |
| 3 | ***GLSZM_GrayLevelNonUniformity-Normalized:*** Scale = 3mm , binWidth = 15 | ***0.02*** | ***FirstOrder_Skewness***  Scale = 4 mm, binWidth = 5 | ***0.002*** |
| 4 | ***GLCM_Imc1;*** Scale = 3mm , binWidth = 10 | ***0.02*** | ***GLSZM_SmallAreaEmphasis:***  Scale = 1 mm, binWidth = 20 | ***0.01*** |
| 5 |  |  | ***GLSZM_SmallAreaLowGrayLevelEmphasis*** Scale = 5mm , binWidth = 20 | ***0.009*** |

**Additional file 1: Table S4: Distant Metastasis (all Variables, p < 0.05)**

| Fx | Gold-Standard SUV Map | p | Simulated SUV Map |  |
| --- | --- | --- | --- | --- |
| 1 | ***GLRLM_ShortRunEmphasis:***  Scale = 2mm , binWidth = 20 | ***0.03*** | ***FirstOrder_Energy:*** Scale = 4mm , binWidth = 5 | ***0.009*** |
| 2 | ***GLSZM_ZoneEntropy***  Scale = 1 mm, binWidth = 20 | ***0.03*** | ***GLSZM_SizeZoneNonUniformityNormalized:*** Scale = 5mm , binWidth = 25 | ***0.02*** |
| 3 | ***GLRLM_GrayLevelNonUniformityNormalized:*** Scale = 1mm , binWidth = 20 | ***0.003*** | ***GLSZM_SmallAreaLowGrayLevelEmphasis***  Scale = 3mm , binWidth = 25 | ***0.014*** |
| 4 | ***GLSZM_GrayLevelNonUniformity;*** Scale = 1mm , binWidth = 25 | ***0.002*** | ***GLCM_InverseVariance*** Scale = 3mm , binWidth = 10 | ***0.007*** |
| 5 |  |  | ***GLSZM_GrayLevelNonUniformity*** Scale = 1mm , binWidth = 25 | ***0.02*** |

**Additional file 1: Table S5: Death (all Variables, p < 0.05)**

| Fx | Gold-Standard SUV Map | p | Simulated SUV Map | p |
| --- | --- | --- | --- | --- |
| 1 | ***GLDM_SmallDependenceEmphasis:***  Scale = 1mm , binWidth = 15 | ***0.01*** | ***GLDM_GrayLevelNonUniformity:*** Scale = 1mm , binWidth = 5 | ***0.001*** |
| 2 | ***GLRLM_GrayLevelNonUniformityNormalized:*** Scale = 1mm , binWidth = 20 | ***<0.001*** | ***GLSZM_SizeZoneNonUniformityNormalized:*** Scale = 5mm , binWidth = 25 | ***0.009*** |
| 3 | ***GLCM_Contrast;*** Scale = 1mm , binWidth = 15 | ***0.0023*** | ***GLSZM_LowGrayLevelZoneEmphasis:*** Scale = 1mm , binWidth = 20 | ***0.04*** |
| 4 | ***GLSZM_GrayLevelNonUniformity;*** Scale = 1mm , binWidth = 25 | ***<0.001*** | ***FirstOrder_Maximum*** Scale = 3mm , binWidth = 5 | ***0.02*** |
| 5 | ***GLSZM_SizeZoneNonUniformityNormalized:*** Scale = 5mm , binWidth = 25 | ***0.03*** | ***GLSZM_SmallAreaEmphasis:***  Scale = 1 mm, binWidth = 5 | ***0.03*** |
| 6 | ***GLSZM_SmallAreaEmphasis:***  Scale = 1 mm, binWidth = 5 | 0.04 |  |  |

**Additional file 1: Table S6: Prediction performance of the best radiomic models from GT/GAN SUV maps (logistic regression)**

|  | *Outcome* | AUC | Sensitivity | Specificity | Accuracy |
| --- | --- | --- | --- | --- | --- |
| GT | ***Locoregional Recurrence*** | 0.60 | 0.67 | 0.55 | 0.59 |
|  | ***Distant Metastasis*** | 0.84 | 0.94 | 0.54 | 0.63 |
|  | ***Death*** | 0.65 | 0.63 | 0.52 | 0.67 |
|  |  |  |  |  |  |
| GAN | ***Locoregional Recurrence*** | 0.59 | 0.62 | 0.70 | 0.59 |
|  | ***Distant Metastasis*** | 0.79 | 0.83 | 0.58 | 0.62 |
|  | ***Death*** | 0.64 | 0.57 | 0.68 | 0.59 |

# References

1. M. Vallieres, E. Kay-Rivest, L. J. Perrin, X. Liem, C. Furstoss, H. Aerts, N. Khaouam, P. F. Nguyen-Tan, C. S. Wang, K. Sultanem, J. Seuntjens, I. El Naqa, Radiomics strategies for risk assessment of tumour failure in head-and-neck cancer. *Sci Rep* **7**, 10117 (2017).

2. P. E. Kinahan, J. W. Fletcher, Positron emission tomography-computed tomography standardized uptake values in clinical practice and assessing response to therapy. *Semin Ultrasound CT MR* **31**, 496-505 (2010).

3. V. Kapoor, B. M. McCook, F. S. Torok, An introduction to PET-CT imaging. *Radiographics* **24**, 523-543 (2004).

4. S. Basu, T. C. Kwee, S. Surti, E. A. Akin, D. Yoo, A. Alavi, Fundamentals of PET and PET/CT imaging. *Ann N Y Acad Sci* **1228**, 1-18 (2011).

5. A. Chandrashekar, A. Handa, P. Lapolla, N. Shivakumar, R. Uberoi, V. Grau, R. Lee, A Deep Learning Approach to Visualise Aortic Aneurysm Morphology without the Use of Intravenous Contrast Agents. Ann Surg, (2021).

6. R. Hong, J. Halama, D. Bova, A. Sethi, B. Emami, Correlation of PET standard uptake value and CT window-level thresholds for target delineation in CT-based radiation treatment planning. *Int J Radiat Oncol Biol Phys* **67**, 720-726 (2007).

7. J. J. M. van Griethuysen, A. Fedorov, C. Parmar, A. Hosny, N. Aucoin, V. Narayan, R. G. H. Beets-Tan, J. C. Fillion-Robin, S. Pieper, H. Aerts, Computational Radiomics System to Decode the Radiographic Phenotype. *Cancer Res* **77**, e104-e107 (2017).

8. M. Radovic, M. Ghalwash, N. Filipovic, Z. Obradovic, Minimum redundancy maximum relevance feature selection approach for temporal gene expression data. *BMC Bioinformatics* **18**, 9 (2017).

9. C. Ding, H. Peng, Minimum redundancy feature selection from microarray gene expression data. *J Bioinform Comput Biol* **3**, 185-205 (2005).
